# Seasonal Changes and Vertical Distribution of Fine Root Biomass During Vegetation Restoration in a Karst Area, Southwest China

**DOI:** 10.3389/fpls.2018.02001

**Published:** 2019-01-11

**Authors:** Hu Du, Lu Liu, Liang Su, Fuping Zeng, Kelin Wang, Wanxia Peng, Hao Zhang, Tongqing Song

**Affiliations:** ^1^Key Laboratory of Agro-Ecological Processes in Subtropical Region, Institute of Subtropical Agriculture, Chinese Academy of Sciences, Changsha, China; ^2^Huanjiang Observation and Research Station for Karst Ecosystems, Institute of Subtropical Agriculture, Chinese Academy of Sciences, Huanjiang, China; ^3^Hunan Agricultural Biotechnology Research Institute, Changsha, China

**Keywords:** seasonal pattern, production, stages of succession, ecological restoration, karst ecosystem

## Abstract

In karst ecosystems, plants absorbing smaller amounts of nutrients, owing to shallow soil, show limited growth. In addition, fine roots (diameter < 2 mm) contribute to the regulation of nutrient cycles in terrestrial ecosystems. However, the spatial and temporal variations of fine root biomass in different vegetation types of the karst region remains poorly understood. In this study, we investigated the seasonal and vertical variation in biomass, necromass, and total mass of fine roots using sequential soil coring under different stages of vegetation restoration (grassland, shrubland, secondary forest, and primary forest) in Southwest China. The results showed that the fine root biomass and necromass ranged from 136.99 to 216.18 g m^−2^ and 47.34 to 86.94 g m^−2^, respectively. The total mass of fine roots and their production ranged from 187.00 to 303.11 g m^−2^ and 55.74 to 100.84 g m^−2^ year^−1^, respectively. They showed a single peak across the vegetation restoration gradient. The fine root biomass and total fine root mass also showed a single peak with seasonal change. In autumn, the fine root biomass was high, whereas the necromass was low. Most of the fine roots were concentrated in the surface soil layer (0–10 cm), which accounted more than 57% root biomass, and decreased with increasing soil depth. In addition, fine root production showed a similar vertical pattern of variation with biomass. Overall, our results suggested that fine roots show clear seasonal and vertical changes with vegetation succession. Moreover, there was a higher seasonal fluctuation and a greater vertical decreasing trend in late-successional stages than in the early-successional stages. The conversion of degraded land to forest could improve the productivity of underground ecosystems and vegetation restoration projects in the fragile karst region should, therefore, continue.

## Introduction

The root biomass is an important part of the biosphere and it constitutes approximately 30% of the aboveground biomass (Yuan and Chen, 2010; Zhiyanski, 2014). Fine roots (<2 mm of diameter) represent a relatively small part of total plant biomass, but they are the most dynamic component of the root systems with highest production and turnover rates (Zhiyanski, 2014; Sun et al., 2015, 2018). They are responsible for water and nutrient uptake, and synthesis of certain growth hormones. They play a vital role in nutrient, water, and elemental cycles (Schmid and Kazda, 2002; McCormack et al., 2017), and in soil carbon sequestration in terrestrial ecosystems, owing to the large carbon input into soil controlled by fine root dynamics (Guo et al., 2007; Wang et al., 2018). High fine root densities increase the hydraulic contact between plants and the soil, thereby increasing water uptake rates and contributing to higher transpiration rates (Gautam and Mandal, 2013). Some studies showed that fine root production is an important component of total net primary production (NPP) in forest ecosystems, contributing 40–60% of total NPP (Zhiyanski, 2014; Meng et al., 2018). Accurate estimates of fine root biomass are essential for understanding ecosystem functions.

Extensive studies have indicated that fine root biomass, necromass, and production vary with vegetation types, and that they vary considerably among the different soil horizons owing to varied water and nutrient content in different soil layers (Hansson et al., 2013; Wang W. et al., 2016; Shu et al., 2018). In most ecosystems, roots tend to be most abundant in the topsoil layer, decreasing exponentially with increasing soil depth (Zhou et al., 2016). Some studies indicated that biomass and productivity are strongly dependent on stand age or developmental stage (Yuan and Chen, 2012; Sun et al., 2015; Pei et al., 2018). Studies have shown that fine roots can show considerable fluctuations in biomass and production throughout the season or among years (Zhiyanski, 2014; Wang P. et al., 2016). Information on the temporal variation in fine root biomass is essential for estimating fine root turnover and production (Fukuzawa et al., 2013). Previous studies have also recorded that fine root biomass is dependent upon soil properties (texture, moisture, chemistry, nutrients) and climate conditions (geographical location, elevation, precipitation, and temperature) (Pei et al., 2018). For example, necromass was higher at both low and high latitudes, whereas less at mid latitudes on a large scale. It was also found to increase with soil organic layer thickness and stand age in a broad-leaf and a needle-leaf forest (Wang et al., 2018). Thus, better knowledge of fine root biomass, productivity, and their spatio-temporal variation induced by vegetation recovery, is essential for taking the long-term carbon dynamics and storage into account.

Karst landscapes are widely distributed globally and comprise almost 18% of the Earth’s surface. Southwest China is one of the largest karst regions in the world, covering about 1.9 million km^2^ (approximately 0.54 million km^2^ of which lies on carbonate rocks) (Du et al., 2017; Brandt et al., 2018). This region is characterized by a high proportion of exposed rock, shallow soil, high soil CaCO_3_ and pH, and specialized regional vegetation (Pan et al., 2016); more than 10,000 years are required to form 1 cm of topsoil in this area (Liu et al., 2016). The ecological systems in this region are extremely fragile and susceptible to land degradation as a result of human disturbance, including intensive mining, deforestation, overgrazing, and overcultivation. Large areas of the karst region in Southwest China were severely degraded following the destruction of natural vegetation and subsequent cultivation (Wen et al., 2016; Li et al., 2018), with a resulting loss of cultivated soil, water shortages, soil erosion, decreased biodiversity, and phyto-community degradation (Tang et al., 2019). The degraded ecosystem seriously threatened local agriculture, forestry, and livestock husbandry. However, most of the degraded land in this region has been undergoing ecological restoration, either through natural regeneration (spontaneous succession) or afforestation, due to the implementation of the “Grain for Green” project and other ecological restoration projects over the past two decades (Lu et al., 2018). Most of the degraded land has seen a shift from cropland or abandoned bare land to forest or other secondary vegetation. Several studies have examined the aboveground biomass in karst regions, including biomass change with vegetation restoration (Cheng et al., 2015; Liu et al., 2016, 2018; Tong et al., 2018). However, our current knowledge of belowground fine root biomass is considerably more limited than that of aboveground biomass in karst regions, and the effects of ecological restoration on fine root biomass have not been evaluated in detail in this fragile ecosystem.

In the present study, we investigated the fine root biomass in a post-agriculture succession sequence, including grassland, shrubland, secondary forest, and primary forest via a space-for-time substitution approach. We aimed to understand the vertical distribution and seasonal pattern of fine root biomass across a vegetation restoration gradient in depressions between karst hills in Southwest China. We hypothesized that fine root biomass would differ among seasons and soil layers according to vegetation restoration types within the karst region.

## Materials and Methods

### Study Area

**FIGURE 1:**
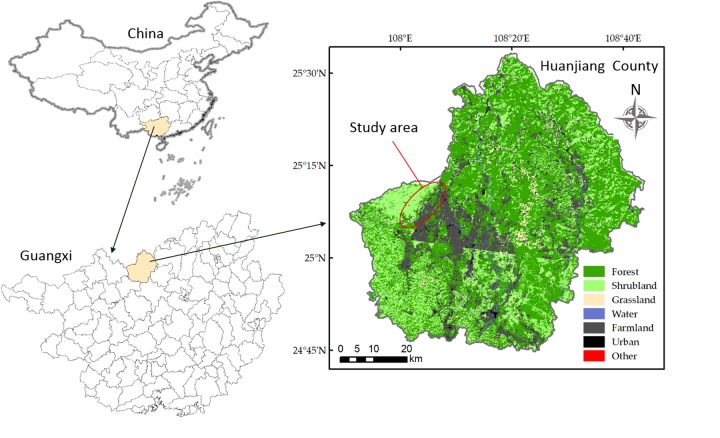
The study area is located in the karst area Southwest China and the vegetation types are shown.

This study was carried out in a karst region of Huanjiang County in northwest Guangxi Zhuang Autonomous Region, Southwest China (Figure 1). A subtropical monsoon climate dominates the study area, with a mean annual precipitation of 1389.1 mm, mean annual cumulative sunshine duration of 1451 h, and mean annual temperature of 15.7°C. The wet season usually lasts from April to September and accounts for about 70% of the total annual precipitation. The coldest month is January, with an average daily temperature of 10.1°C, and the hottest month is July, with an average daily temperature of 28.0°C. The mean annual frost-free period lasts for 290 days. The area has a mean annual evaporation of 1571.1 mm and 70% relative humidity (Hu et al., 2017). The region is characterized by a typical karst landscape with gentle valleys flanked by steep hills. The soil is a calcareous lithosol (limestone soil) (Wen et al., 2017). Soil pH and soil organic carbon varied from 7.06 to 7.68 and 53.96 to 82.63 g kg^−1^, respectively. Total N, total P, total K, available N, available P, and available K ranged from 6.65 to 9.85 g kg^−1^, 0.89 to 1.98 g kg^−1^, 3.05 to 4.69 g kg^−1^, 257.95 to 618.67 mg kg^−1^, 1.84 to 14.05 mg kg^−1^, and 1.79 to 6.09 mg kg^−1^, respectively (Table 1). These soil chemical properties were measured referring to Hu et al. (2017).

**Table 1:** Community characteristics and soil properties (0–10 cm) in the four vegetation types.

Characteristic	Vegetation types			
	Grassland	Shrubland	Secondary forest	Primary forest
Tree Density (tree hm^−2^)	–	5608	4625	4433
Mean height (m)	0.66	2.84	4.96	6.37
Mean DBH (cm)	–	2.45	4.69	6.16
Soil organic carbon (g kg^−1^)	53.96	63.13	71.38	82.63
Soil total N (g kg^−1^)	8.72	9.85	7.20	6.65
Soil total P (g kg^−1^)	0.89	1.14	1.98	1.60
Soil total K (g kg^−1^)	4.33	4.69	4.11	3.05
Soil available N (mg kg^−1^)	257.95	354.60	484.91	618.67
Soil available P (mg kg^−1^)	2.63	1.84	8.03	14.05
Soil available K (mg kg^−1^)	1.79	4.03	2.92	6.09
Soil pH value	7.34	7.06	7.35	7.68

In recent decades, forests have been largely destroyed by human disturbance, resulting in diverse vegetation types in this region (Du et al., 2014; Wen et al., 2016). A post-agriculture succession sequence, including four vegetation types, i.e., grassland, shrubland, secondary forest, and primary forest, was selected based on a space-for-time substitution approach. The stand characteristics and soil properties are presented in Table 1.

### Experimental Design

In June 2016, we used a randomized complete block design with three blocks. Each block was more than 2 km away from the next block. The block was a strip region from agricultural activity area to nature reserve. Many vegetation types in various successional stages were distributed in the block. Within each block, one plot (20 m × 20 m) was established for each of the four vegetation types, and the distances between plots were more than 200 m (see Supplementary Figure S1 for details). The major species found in the grassland included *Imperata cylindrica*, *Microstegium fasciculatum*, and *Murdannia triquetra*. The major species found in the shrubland included *Pyracantha fortuneana*, *Vitex negundo*, and *Alchornea trewioides*. The secondary forest was mainly composed of *Cryptocarya microcarpa*, *Itoa orientalis*, and *Litsea lancifolia*. The primary forest was composed mainly of *Cyclobalanopsis glauca*, *Platycarya longipes*, and *Handeliodendron bodinieri*.

### Fine Root Sampling and Processing

In this study, a sequential soil coring technique was used to estimate fine root biomass, necromass, and fine root production of the four vegetation stands (Makkonen and Helmisaari, 1999; Brunner et al., 2013). Root sampling was carried out from June 2016 to May 2017. Eight soil cores in each plot were randomly collected each month from each plot. The soil cores were collected using a steel soil corer with a diameter of 10 cm to a soil depth of 30 cm. The soil cores were divided into three different soil depths: 0–10, 10–20, and 20–30 cm. There were 36 samples in total (4 vegetation types × 3 replicate plots × 3 soil depths) at each sampling time. All the samples were transferred into plastic bags, transported, and placed in a refrigerator at 4°C until later processing (Sun et al., 2015).

In the laboratory, fine root samples (<2 mm) were washed to free them from adhering soil and organic matter, and separated manually into living roots (biomass) and dead roots (necromass) based on visual inspection, described by Vogt and Persson (1991). Living roots were elastic, flexible, and the stele was bright to slightly brown. In contrast, dead roots were easily broken, with brown or black steles. Thereafter, the sorted samples were dried at 70°C to a constant mass and weighed. The fine root biomass (g m^−2^) in each soil layer was equal to the dry mass (g) divided by the sectional area (m^2^) of eight steel soil corers.

### Calculations and Statistical Analysis

Fine root biomass and necromass were estimated using the collected samples from the sequential soil coring technique during the one-year measurement period in different seasons (Spring: March–May; Summer: June–August; Autumn: September–November; Winter: December–February). The root mass of a season was the average mass in 3 months. In addition, fine root production was estimated from sequential soil cores and calculated by the balancing the living and dead fine root mass compartments according the method of the Decision Matrix (DM). The production between two continuous seasons was calculated either by adding the differences in biomass and necromass, by adding only the differences in biomass, or by equalling production to zero depending on the relative changes of biomass and necromass (Xiong et al., 2017). Detailed descriptions of the DM can be found in Brunner et al. (2013).

Multi-way analysis of variance (ANOVA) was used to examine the effect of vegetation type, season, soil depth and block on fine root biomass, necromass, and total fine root (living + dead) mass. Differences in biomass among vegetation types, seasons, or soil layers were determined by one-way ANOVA followed by the Tukey test. Differences in production among soil layers and vegetation types were also determined by one-way ANOVA followed by the Tukey test. Log transformation were conducted prior to analysis in order to meet ANOVA requirements for homogeneity of variance (Xiong et al., 2017). All the statistical analyses were performed using the R3.3.2 software (R Core Team, 2016). In all case, the accepted significance level was α = 0.05.

## Results

**FIGURE 2:**
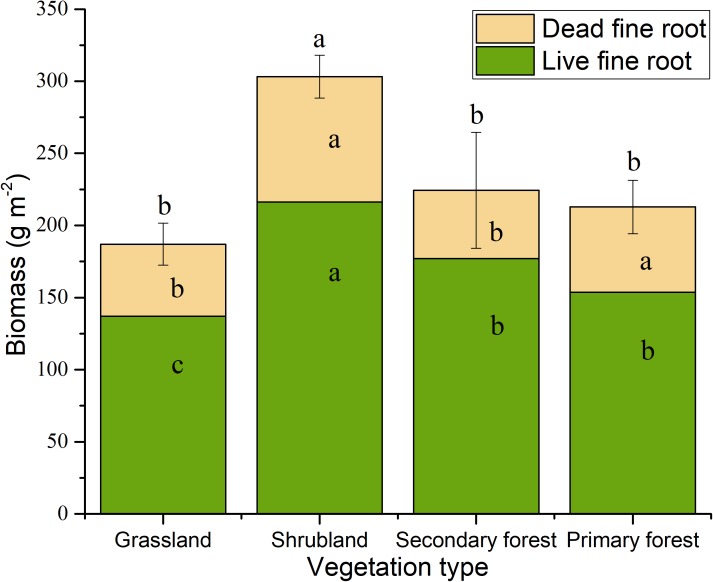
Fine root biomass in the four vegetation restoration stages. The value is the average biomass across four seasons. Error bars represent standard deviation of total biomass. Different letters indicate significant differences between vegetation types (*p* < 0.05). Letters in lower position represent live fine root, letters in middle position represent dead fine root, letters in upper position represent total mass.

We found that the vegetation type, soil layer, and season have significant effects on fine root biomass and necromass, and that the effects of the interactions of vegetation type and soil layer were significant (Table 2). The total fine root mass (0–30 cm depth) was 187.00 g m^−2^ in grasslands, 303.11 g m^−2^ in shrublands, 224.28 g m^−2^ in secondary forests, and 212.84 g m^−2^ in primary forests, respectively. The standing fine root biomass showed higher values than fine root necromass in different stages of vegetation restoration (Figure 2).

**Table 2:** Effects of vegetation, season, soil layer, and block on fine root biomass and necromass using analysis of variance.

		Fine root biomass			Fine root necromass		
Source	df	Sum of squares	*F*-value	*P*-value	Sum of squares	*F*-value	*P*-value
Block	2	0.23	0.731	0.4839	0.17	0.666	0.516
Vegetation	3	15.03	32.130	<***0.001***	19.02	49.828	<***0.001***
Season	3	1.56	3.340	***0.023***	7.85	20.571	<***0.001***
Layer	2	93.36	299.315	<***0.001***	55.70	218.899	<***0.001***
Vegetation × season	9	0.83	0.594	0.797	1.53	1.333	0.225
Vegetation × layer	6	15.80	16.889	<***0.001***	3.79	4.965	<***0.001***
Season × layer	6	1.43	1.533	0.173	1.28	1.680	0.136
Vegetation × season × layer	18	2.25	0.800	0.691	3.10	1.354	0.168

*Data were ln(x + 1) transformed, italicized and bold fonts indicate significant differences at p < 0.05.*

**FIGURE 3:**
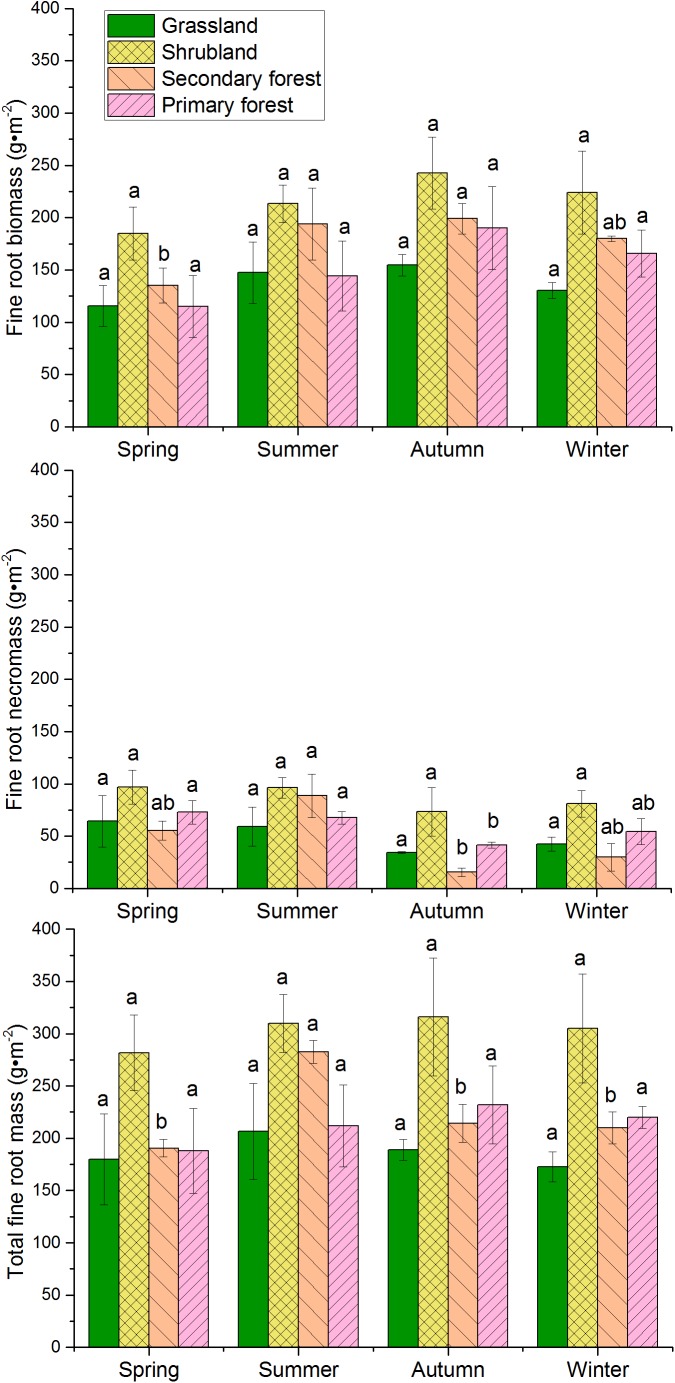
Seasonal variations in fine root biomass, necromass, and total mass in the four vegetation restoration stages. Error bars represent standard deviation. Different letters represent significant differences among seasons within each vegetation type (*p* < 0.05).

Fine root biomass, necromass, and total fine root mass (live + dead) varied seasonally (Figure 3). Fine root biomass of all the four vegetation types increased from spring, peaked in autumn, and declined in winter. However, fine root necromass showed the lowest value in autumn. The necromass decreased from spring to autumn, and then increased from autumn to winter in grasslands, shrublands, and primary forests. The seasonal change in total fine root mass showed a single peak. The peak value occurred during summer in grasslands and secondary forests, whereas it occurred during autumn in shrublands and primary forests.

**FIGURE 4:**
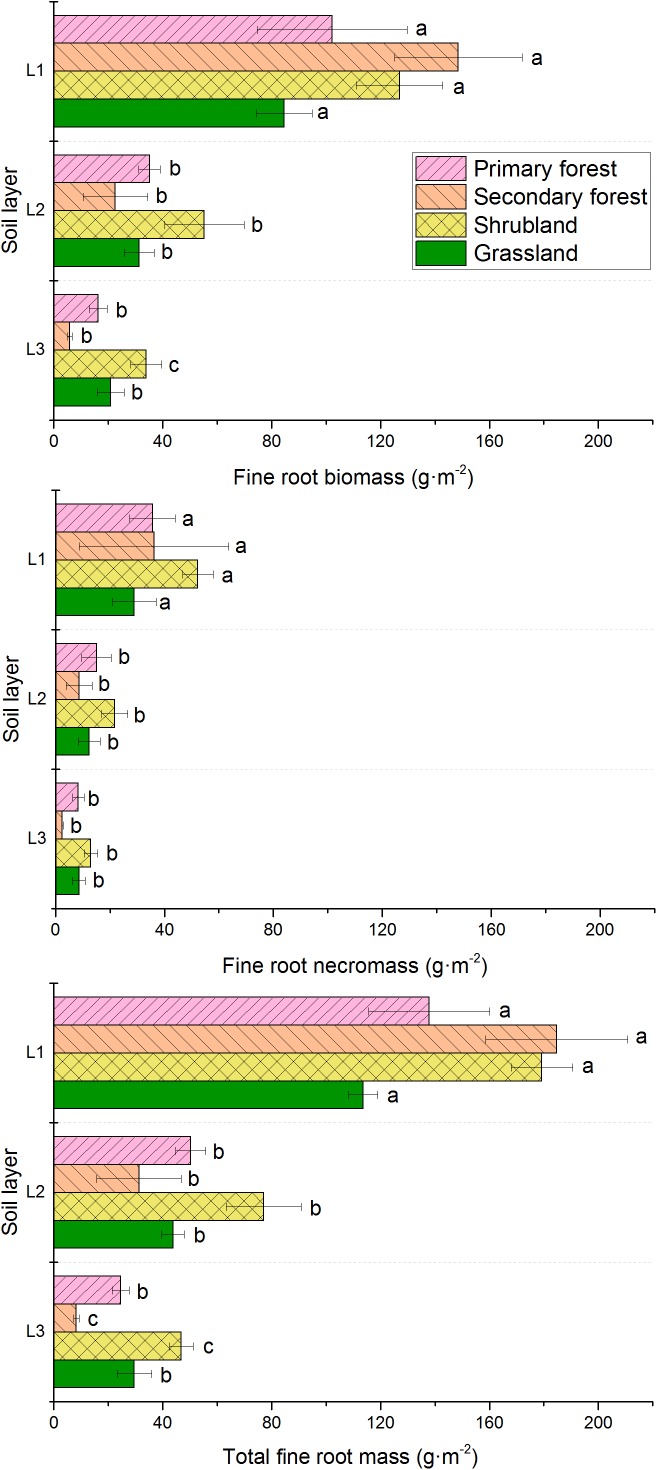
Vertical distribution of fine root biomass, necromass, and total mass in the four vegetation restoration stages. L1, 0–10 cm soil layer; L2, 10–20 cm soil layer; L3, 20–30 cm soil layer. Error bars represent standard deviation. Different letters indicate significant differences among soil layers within each vegetation type (*p* < 0.05).

Fine root biomass, necromass, and total mass were concentrated to the surface soil (0–10 cm depth) and decreased with increasing soil depth (Figure 4). The biomass in the 0–10 cm soil layer were significantly higher than that in other soil layers, respectively. The vertical distribution of fine roots differed among the four vegetation types. In the uppermost soil layer, the fine root biomass and total fine root mass could be ordered as follows: secondary forests > primary forests > shrublands > grasslands. However, in the 10–20 and 20–30 cm soil layers, the secondary forests had the lowest biomass. Necromass in the shrublands in all the three soil layers were higher than that in the other vegetation types. In addition, similar patterns were found in total fine root mass in the 10–20 and 20–30 cm soil layers.

**FIGURE 5:**
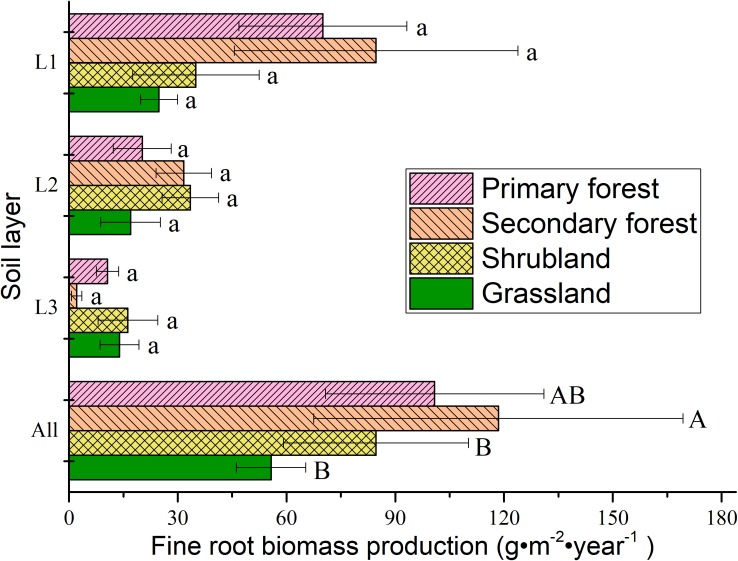
Fine root production in different soil layers in the four vegetation types. L1, 0–10 cm soil layer; L2, 10–20 cm soil layer; L3, 20–30 cm soil layer; All, 0–30 cm soil layer. Error bars represent standard deviation. Different lowercase letters indicate significant differences among soil layers within each vegetation type (*p* < 0.05). Different capital letters indicate significant differences between vegetation types (*p* < 0.05).

Annual fine root production ranged between 55.74 and 100.84 g m^−2^ year^−1^ across the soil layers (i.e., to a depth of 30 cm), and it could be ordered as follows: secondary forests > primary forests > shrublands > grasslands (Figure 5). The production in the surface layer (0–10 cm) accounted for 41.30–71.51% of the production in all the soil layers, and the production decreased with increasing soil depth.

## Discussion

Trends in aboveground biomass along vegetation restoration gradient or stand age have been extensively studied (Liu et al., 2016, 2015; Zhang et al., 2017). However, few studies have been carried out on the changes in belowground biomass along vegetation recovery or succession stages. In addition, quantification of fine root biomass is significant for belowground ecosystems due to its crucial role in soil organic matter accumulation and nutrient absorption (Meng et al., 2018). In the present study, the fine root biomass, necromass, the total fine root mass, and fine root production were estimated along a post-agriculture succession sequence in a karst region Southwest China. The results showed that fine root dynamics change with vegetation succession. Fine root biomass, necromass, total fine root mass was the largest in shrublands. The successional changes in tree density with stand development could contribute to the observed patterns. In this study, the fine root biomass of secondary forests and primary forests in the 0–30 cm soil layer was lower than that in non-karst communities in a subtropical region (Hunan Province) that included 10- and 24-year-old mixed plantations of *Pinus massoniana* and *Cinnamomum camphora* (Shu et al., 2018), but similar to that of the 45-year-old stands. Moreover, the fine root biomass of karst vegetation in Maolan, bordering our study area, show similar results (Ni et al., 2015). Consistent with our findings, the change in fine root biomass and production have been reported to peak in middle stage of succession (Figures 2, 5). For example, Yuan and Chen (2012) showed that the fine root production increased with stand development, and then declined. Sun et al. (2015) found that intermediate-aged stand had the highest fine root biomass across a *Betula platyphylla* chronosequence.

Fine roots constitute a very dynamic part of the root systems, which are usually responsive to changes in soil temperature, moisture, and nutrient content (Liu et al., 2014; Xiong et al., 2017). In the present study, the fine root biomass, necromass, and total fine root mass in the four vegetation restoration stages showed different seasonal variation patterns that increased or decreased at different points in time. These results could be due to differences in the microclimate in the understory (water availability, soil temperature), community phenological patterns, or phyto-community diversity (Fukuzawa et al., 2007; Xiong et al., 2017). The fine root biomass increased in spring, peaked from summer to autumn, and decreased in winter. Another study demonstrated that the maximum fine root biomass occurred in autumn owing to fine root production during the growing season (Brassard et al., 2009; Hansson et al., 2013). In the forest ecosystems, the fine root biomass showed a relatively high seasonal fluctuation (i.e., a higher variable coefficient in primary and secondary forests than in shrublands and grasslands). This is likely due to the responses to the greater species richness in forests than in shrublands and grasslands, which was shown in our previous study (Du et al., 2013; Hu et al., 2017). It suggested that species-rich forests possess a higher resistance capacity under environmental stress, such as soil water and nutrients. In the present study, fine root necromass in the four vegetation types exhibited different tendencies with seasonal changes in fine root biomass, and the lowest abundance of necromass among all the vegetation types were found in autumn. This result could be attributed to higher temperature and decreased soil moisture in summer, and to low temperature in winter (Konôpka et al., 2006). Konôpka et al. (2006) concluded that necromass in mid-growing season was significantly less than that in the beginning and end of the growing season. The seasonality of fine root mass suggested that root sampling in different seasons is necessary to obtain a complete overview of the dynamic of fine root biomass.

Our findings showed that the fine root biomass, necromass, and total fine root mass in the four vegetation types decreased with soil depth (Figures 4, 5). More than 57% of the mass was confined to uppermost 10 cm of the soil layer. The high density of fine roots within a few centimeters from the soil surface is crucial for acquiring nutrients. Several studies demonstrate the decreasing trend in the soil profile (Fukuzawa et al., 2007; Yuan and Chen, 2010; Liu et al., 2014; Sun et al., 2015). Although fine root biomass of all the vegetation types decreased with soil depth, the vertical live fine root-distribution patterns were not the same. The vertical distribution of fine root biomass decreased more sharply in forests (especially in the secondary forests) than in the other vegetation types. Moreover, Liu et al. (2014) suggested that the vertical distribution patterns of fine roots decreased more sharply in the species-rich community than in the species-poor community. In our study area, we also found more plant species in forests (128 species in primary forest, 153 species in secondary forest) than in other vegetation types (87 species in shrubland, 26 species in grassland) (Hu et al., 2017). In the present study, the order of fine root biomass proportion in the deeper layer (10–30 cm) could be ordered as follows: shrubland > grassland > primary forest > secondary forest, which suggested that root systems of early-successional communities were more effective in exploring nutrients and water in deep soils. Yuan and Chen (2010) also found that early-successional species had higher proportion of roots in deeper soil layers than the late-successional species had. In addition, the production of fine roots decreased with increasing soil depth (Figure 5), and similar vertical distributions were observed in numerous previous studies (Fukuzawa et al., 2007; Yuan and Chen, 2012; Hansson et al., 2013; Sun et al., 2015). This distribution pattern may result from the change in soil water content, nutrient content, and bulk density in the soil profile (Fukuzawa et al., 2007; Ostonen et al., 2011; Sun et al., 2015). Looking to the future, we recommend additional studies that explore individual non-linear models to better understand changes in the fine root biomass over time and the factors involved, as shown for aerial biomass in previous studies (Girona et al., 2017).

## Conclusion

The present study elucidated the vertical distribution and seasonal patterns of fine root biomass along vegetation restoration gradients in karst areas of Southwest China. Our results showed significant effects of vegetation restoration stages, seasons, and soil layers on fine root biomass and necromass. The dynamics of fine root biomass, necromass, and total fine root mass peaked in shrublands. The fine root biomass peaked in autumn, when the lowest fine root necromass was observed. High fine root biomass found in the upper soil layer, showed a continuous decrease with soil depth in all the vegetation types studied. Fine root production showed similar vertical patterns as that of fine root biomass, and the former showed a single peak during the vegetation restoration process. Overall, knowledge of spatiotemporal patterns of the dynamics of root systems contribute to our understanding of underground processes, which might help in evaluating the carbon cycle in the karst area studied. In karst regions, the conversion of degraded land to forest can effectively improve the productivity of underground ecosystems, and greater attention should be paid to the upper soil layers. However, the optimum status of vegetation restoration (community structure and diversity) for improving underground ecological function needs further elucidation.

## Author Contributions

HD, FZ, and TS designed the study. HD and LS performed the experiments, collected and analyzed the data. HD and LL wrote the manuscript. KW, WP, and HZ discussed the experimental design and manuscript writing.

## Conflict of Interest Statement

The authors declare that the research was conducted in the absence of any commercial or financial relationships that could be construed as a potential conflict of interest.

## References

[B1] Brandt M. Yue Y. M. Wigneron J. P. Tong X. W. Tian F. Jepsen M. R. (2018). Satellite-observed major greening and biomass increase in south china karst during recent decade. *Earths Future* 6 1017–1028. 10.1029/2018EF000890

[B2] Brassard B. W. Chen H. Y. H. Bergeron Y. (2009). Influence of environmental variability on root dynamics in northern forests. *Crit. Rev. Plant Sci.* 28 179–197. 10.1080/07352680902776572

[B3] Brunner I. Bakker M. R. Björk R. G. Hirano Y. Lukac M. Arana X. (2013). Fine-root turnover rates of European forests revisited: an analysis of data from sequential coring and ingrowth cores. *Plant Soil* 362 357–372. 10.1007/s11104-012-1313-5

[B4] Cheng J. Lee X. Theng B. K. G. Zhang L. Fang B. Li F. S. (2015). Biomass accumulation and carbon sequestration in an age-sequence of *Zanthoxylum bungeanum* plantations under the grain for green program in karst regions, guizhou province. *Agric. For. Meteorol.* 203 88–95. 10.1016/j.agrformet.2015.01.004

[B5] Du H. Hu F. Zeng F. P. Wang K. L. Peng W. X. Zhang H. (2017). Spatial distribution of tree species in evergreen-deciduous broadleaf karst forests in southwest China. *Sci. Rep.* 7:15664. 10.1038/s41598-017-15789-5 29142282 PMC5688135

[B6] Du H. Peng W. X. Song T. Q. Wang K. L. Zeng F. P. Lu S. Y. (2013). Plant community characteristics and its coupling relationships with soil in depressions between karst hills, North Guangxi, China. *Chin. J. Plant Ecol.* 37 197–208. 10.3724/SP.J.1258.2013.00020

[B7] Du H. Wang K. L. Peng W. X. Zeng F. P. Song T. Q. Zhang H. (2014). Spatial heterogeneity of soil mineral oxide components in depression between karst hills, Southwest China. *Chin. Geogr. Sci.* 24 163–179. 10.1007/s11769-013-0630-9

[B8] Fukuzawa K. Shibata H. Takagi K. Satoh F. Koike T. Sasa K. (2007). Vertical distribution and seasonal pattern of fine-root dynamics in a cool–temperate forest in northern Japan: implication of the understory vegetation, sasa dwarf bamboo. *Ecol. Res.* 22 485–495. 10.1007/s11284-006-0031-y

[B9] Fukuzawa K. Shibata H. Takagi K. Satoh F. Koike T. Sasa K. (2013). Temporal variation in fine-root biomass, production and mortality in a cool temperate forest covered with dense understory vegetation in northern Japan. *For. Ecol. Manage.* 310 700–710. 10.1016/j.foreco.2013.09.015

[B10] Gautam T. P. Mandal T. N. (2013). Effect of disturbance on fine root biomass in the tropical moist forest of eastern Nepal. *Nepal. J. Biosci.* 2 10–16. 10.3126/njbs.v2i0.7484

[B11] Girona M. M. Rossi S. Lussier J. M. Walsh D. Morin H. (2017). Understanding tree growth responses after partial cuttings: a new approach. *PloS One.* 12:e0172653. 10.1371/journal.pone.0172653 28222200 PMC5319695

[B12] Guo L. B. Wang M. Gifford R. M. (2007). The change of soil carbon stocks and fine root dynamics after land use change from a native pasture to a pine plantation. *Plant Soil* 299 251–262. 10.1007/s11104-007-9381-7

[B13] Hansson K. Helmisaari H. S. Sah S. P. Lange H. (2013). Fine root production and turnover of tree and understorey vegetation in Scots pine, silver birch and Norway spruce stands in SW Sweden. *For. Ecol. Manage.* 309 58–65. 10.1016/j.foreco.2013.01.022

[B14] Hu F. Du H. Zeng F. P. Peng W. X. Song T. Q. (2017). Plant community characteristics and their relationships with soil properties in a karst region of southwest China. *Contemp. Probl. Ecol.* 10 707–716. 10.1134/S1995425517060051

[B15] Konôpka B. Noguchi K. Sakata T. Takahashi M. Konôpkováb Z. (2006). Fine root dynamics in a Japanese cedar (*Cryptomeria japonica*) plantation throughout the growing season. *For. Ecol. Manage.* 225 278–286. 10.1016/j.foreco.2006.01.004

[B16] Li D. Wen L. Jiang S. Song T. Q. Wang K. L. (2018). Responses of soil nutrients and microbial communities to three restoration strategies in a karst area, southwest China. *J. Environ. Manage.* 207 456–464. 10.1016/j.jenvman.2017.11.067 29197267

[B17] Liu B. Zhao W. Z. Liu Z. L. Yang Y. T. Luo W. C. Zhou H. (2015). Changes in species diversity, aboveground biomass, and vegetation cover along an afforestation successional gradient in a semiarid desert steppe of China. *Ecol. Eng.* 81 301–311. 10.1016/j.ecoleng.2015.04.014

[B18] Liu C. Xiang W. H. Lei P. F. Deng X. W. Tian D. L. Fang X. (2014). Standing fine root mass and production in four Chinese subtropical forests along a succession and species diversity gradient. *Plant Soil* 376 445–459. 10.1007/s11104-013-1998-0

[B19] Liu C. C. Liu Y. G. Guo K. Wang S. J. Liu H. M. Zhao H. W. (2016). Aboveground carbon stock, allocation and sequestration potential during vegetation recovery in the karst region of southwestern China: a case study at a watershed scale. *Agric. Ecosyst. Environ.* 235 91–100. 10.1016/j.agee.2016.10.003

[B20] Liu L. B. Ni J. Zhong Q. L. Hu G. Zhang Z. H. (2018). High mortality and low net change in live woody biomass of karst evergreen and deciduous broad-leaved mixed forest in southwestern China. *Forests* 9:263 10.3390/f9050263

[B21] Lu F. Hu H. Sun W. Zhu J. Liu G. Zhou W. (2018). Effects of national ecological restoration projects on carbon sequestration in China from 2001 to 2010. *Proc. Natl. Acad. Sci. U.S.A.* 115 4039–4044. 10.1073/pnas.1700294115 29666317 PMC5910802

[B22] Makkonen K. Helmisaari H. S. (1999). Assessing fine-root biomass and production in a Scots pine stand–comparison of soil core and root ingrowth core methods. *Plant Soil* 210 43–50. 10.1023/A:1004629212604

[B23] McCormack M. L. Guo D. L. Iversen C. M. Chen W. L. Eissenstat D. M. Fernandez C. W. (2017). Building a better foundation: improving root-trait measurements to understand and model plant and ecosystem processes. *New Phytol.* 215 27–37. 10.1111/nph.14459 28295373

[B24] Meng S. W. Jia Q. Q. Zhou G. Zhou H. Liu Q. J. Yu J. (2018). Fine root biomass and its relationship with aboveground traits of *Larix gmelinii* trees in northeastern China. *Forests* 9:35 10.3390/f9010035

[B25] Ni J. Luo D. H. Xia J. Zhang Z. H. Hu G. (2015). Vegetation in karst terrain of southwestern China allocates more biomass to roots. *Solid Earth* 6 799–810. 10.5194/se-6-799-2015

[B26] Ostonen I. Helmisaari H. S. Borken W. Tedersoo L. Kukumägi M. Bahram M. (2011). Fine root foraging strategies in Norway spruce forests across a European climate gradient. *Glob. Chang. Biol.* 17 3620–3632. 10.1111/j.1365-2486.2011.02501.x

[B27] Pan F. J. Liang Y. M. Zhang W. Zhao J. Wang K. (2016). Enhanced nitrogen availability in karst ecosystems by oxalic acid release in the rhizosphere. *Front. Plant Sci.* 7:687. 10.3389/fpls.2016.00687 27252713 PMC4877511

[B28] Pei Y. M. Lei P. F. Xiang W. H. Ouyang S. Xu Y. Y. (2018). Effect of stand age on fine root biomass, production and morphology in Chinese fir plantations in subtropical China. *Sustainability* 10:2280 10.3390/su10072280

[B29] R Core Team. (2016). *R: A Language and Environment for Statistical Computing*. Vienna: R Foundation for Statistical Computing.

[B30] Schmid I. Kazda M. (2002). Root distribution of Norway spruce in monospecific and mixed stands on different soils. *For. Ecol. Manage.* 159 37–47. 10.1016/S0378-1127(01)00708-3

[B31] Shu W. W. Shen X. X. Lei P. F. Xiang W. H. Ouyang S. Yan W. D. (2018). Temporal changes of fine root overyielding and foraging strategies in planted monoculture and mixed forests. *BMC Ecol.* 18:9. 10.1186/s12898-018-0166-z 29454355 PMC5816503

[B32] Sun T. Dong L. L. Mao Z. J. Li Y. Y. (2015). Fine root dynamics of trees and understorey vegetation in a chronosequence of *Betula platyphylla* stands. *For. Ecol. Manage.* 346 1–9. 10.1016/j.foreco.2015.02.035

[B33] Sun T. Hobbie S. E. Berg B. Zhang H. G. Wang Q. K. Wang Z. W. (2018). Contrasting dynamics and trait controls in first-order root compared with leaf litter decomposition. *Proc. Natl. Acad. Sci. U.S.A.* 115 10392–10397. 10.1073/pnas.1716595115 30254167 PMC6187159

[B34] Tang J. Tang X. X. Qin Y. M. He Q. S. Yi Y. Ji Z. L. (2019). Karst rocky desertification progress: soil calcium as a possible driving force. *Sci. Total Environ.* 649 1250–1259. 10.1016/j.scitotenv.2018.08.242 30308895

[B35] Tong X. W. Brandt M. Yue Y. M. Horion S. Wang K. L. Keersmaecker W. D. (2018). Increased vegetation growth and carbon stock in China karst via ecological engineering. *Nat. Sustain.* 1 44–50. 10.1038/s41893-017-0004-x

[B36] Vogt K. A. Persson H. A. (1991). “Measuring growth and development of roots,” in *Techniques and Approaches in Forest Tree Ecophysiology*, eds Lassoie J. P. Hinckley T. M. (New York, NY: CRS Press), 477–501.

[B37] Wang C. G. Chen Z. Brunner I. Zhang Z. Zhu X. J. Li J. D. (2018). Global patterns of dead fine root stocks in forest ecosystems. *J. Biogeogr.* 45 1378–1394. 10.1111/jbi.13206

[B38] Wang P. Mommerk L. van Ruijvenk J. Erendse F. Maximov T. C. Heijmans M. (2016). Seasonal changes and vertical distribution of root standing biomass of graminoids and shrubs at a Siberian tundra site. *Plant Soil* 407 55–65. 10.1007/s11104-016-2858-5

[B39] Wang W. Wu X. G. Hu K. Liu J. C. Tao J. P. (2016). Understorey fine root mass and morphology in the litter and upper soil layers of three Chinese subtropical forests. *Plant Soil* 406 219–230. 10.1007/s11104-016-2878-1

[B40] Wen L. Li D. J. Chen H. Wang K. L. (2017). Dynamics of soil organic carbon in density fractions during post-agricultural succession over two lithology types, southwest China. *J. Environ. Manage.* 201 199–206. 10.1016/j.jenvman.2017.06.048 28666196

[B41] Wen L. Li D. J. Yang L. Q. Luo P. Chen H. Xiao K. C. (2016). Rapid recuperation of soil nitrogen following agricultural abandonment in a karst area, southwest China. *Biogeochemistry* 129 341–354. 10.1007/s10533-016-0235-3

[B42] Xiong Y. M. Liu X. Guan W. Liao B. W. Chen Y. J. Li M. (2017). Fine root functional group based estimates of fine root production and turnover rate in natural mangrove forests. *Plant Soil* 413 83–95. 10.1007/s11104-016-3082-z

[B43] Yuan Z. Y. Chen H. Y. H. (2010). Fine root biomass, production, turnover rates, and nutrient contents in boreal forest ecosystems in relation to species, climate, fertility, and stand age: literature review and meta-analyses. *Crit. Rev. Plant Sci.* 29 204–221. 10.1080/07352689.2010.483579

[B44] Yuan Z. Y. Chen H. Y. H. (2012). Fine root dynamics with stand development in the boreal forest. *Funct. Ecol.* 26 991–998. 10.1111/j.1365-2435.2012.02007.x

[B45] Zhang H. Wang K. L. Zeng Z. X. Du H. Zeng F. P. (2017). Biomass and carbon sequestration by *Juglans regia* plantations in the karst regions of southwest China. *Forests* 8:103 10.3390/f8040103

[B46] Zhiyanski M. (2014). Seasonal dynamics of fine root biomass in selected forest stands. *Silva Balcanica* 15 5–15.

[B47] Zhou H. Zhao W. Z. Yang Q. Y. (2016). Root biomass distribution of planted *Haloxylon ammodendron* in a duplex soil in an oasis: desert boundary area. *Ecol. Res.* 31 673–681. 10.1007/s11284-016-1376-5

