# Supplementary material for: Seasonal Changes and Vertical Distribution of Fine Root Biomass During Vegetation Restoration in a Karst Area, Southwest China
Source: Front Plant Sci. 2019 Jan 11;9:2001. doi: 10.3389/fpls.2018.02001 (PMC6337902; doi:10.3389/fpls.2018.02001)
Supplement: Supplementary file 1 [file Table_1.DOC]

**Seasonal changes and vertical distribution of fine root biomass during vegetation restoration in a karst area, southwest China**

Hu Du^1,2^, Lu Liu^3^, Liang Su^1,2^, Fuping Zeng^1,2^, Kelin Wang^1,2^, Wanxia Peng^1,2^, Hao Zhang^1,2^, & Tongqing Song^1,2*^

^1^Key Laboratory of Agro-ecological Processes in Subtropical Region, Institute of Subtropical Agriculture, Chinese Academy of Sciences, Changsha 410125, Hunan, China

^2^Huanjiang Observation and Research Station for Karst Ecosystems, Institute of Subtropical Agriculture, Chinese Academy of Sciences, Huanjiang 547100, Guangxi, China

^3^Hunan Agricultural Biotechnology Research Institute, Changsha 410125, Hunan, China

* Correspondence: Tongqing Song Email: songtongq@isa.ac.cn

Block 1

Block 2

Block 3

GL

SL

SF

PF

SL

SF

PF

GL

PF

GL

SL

SF

20 m

20 m

Figure S1 The schematic representation of Experimental design. GL, grassland; SL, shrubland; SF, secondary forest; PF, primary forest.
